# Structural biology at the National Synchrotron Light Source II

**DOI:** 10.1107/S1600577525003194

**Published:** 2025-06-26

**Authors:** J. Aishima, B. Andi, L. Berman, J. Byrnes, S. Chodankar, E. Farquhar, M. R. Fuchs, J. Jakoncic, D. Kreitler, E. Lazo, S. Myers, K. Qian, R. Schaffer, V. Shekar, W. Shi, A. Soares, V. Stojanoff, R. M. Sweet, L. Yang, S. McSweeney

**Affiliations:** a National Synchrotron Light Source II Brookhaven National Laboratory Upton NY USA; b Case Western Reserve University, Cleveland, OH, USA; University of Manchester, United Kingdom

**Keywords:** structural biology, National Synchrotron Light Source II, research resources, synchrotron beamlines

## Abstract

The structural biology program at the National Synchrotron Light Source II presents a coordinated set of instruments, software and research opportunities for the interested user. We describe in some detail the research capabilities enabled by the Center for BioMolecular Structure. The evolution of the resources is described in detail, considering three major themes: automation, micro-focusing and computation prediction.

## Abstract 2

We describe the structural biology resources available at the National Synchrotron Light Source II at Brookhaven National Laboratory and ponder the future for automated experiments, micro-focusing crystallography and structure prediction to inform structural biology studies.

## 1. Introduction

Structural biology stands at an exciting point in its history. The new generations of light sources provide exquisitely bright, high-flux beamlines that open new research directions for structural biology. Cryogenic electron microscopy has advanced to such a degree that it is an essential part of the tool kit for structural biology. An important new tool is the capability of protein structure modeling through the use of artificial intelligence/machine learning (AI/ML). At Brook­haven National Laboratory (BNL) we are embracing the opportunity presented by the synergy of electron and X-ray techniques. We have established an electron cryo-microscopy capability to work in partnership with the efforts described here using the National Synchrotron Light Source II (NSLS-II). With support from both the National Institutes of Health (NIH) and the Department of Energy, through the National Institute of General Medical Sciences (NIGMS) and Biological and Environmental Research program, support for structural biology is coordinated under the auspices of the Center for BioMolecular Structure (CBMS) (https://www.bnl.gov/nsls2/lifesciences).

The first light was observed at NSLS-II, a little over a decade ago in October of 2014. Construction of the structural biology beamlines that form the basis of this report was underway at that time, with the first four instruments delivering photons between October 2016 and April 2018. The macromolecular crystallography (MX) beamlines at 17-ID-1 and 17-ID-2 (Highly Automated MX; and Frontier MX, AMX and FMX, respectively) have enabled routine, automated microfocused X-ray crystallography (Fuchs *et al.*, 2016 ▸; Schneider *et al.*, 2022 ▸). Automated diffraction data collection (for crystals >20 µm) and use of X-ray focused beams (routinely <2 µm × 2 µm) were made available. We have created a multipurpose X-ray scattering facility for life sciences at 16-ID: the beamline that provides small-angle protein solution scattering (both automated static scattering and after size-exclusion chromatography) (Yang *et al.*, 2020 ▸) along with imaging methods (Yang *et al.*, 2022 ▸). The synchrotron X-ray footprinting (XFP) beamline is run by partners at Case Western Reserve University and provides a mature synchrotron technique complementary to the resources available within CBMS.

Access to these CBMS resources is coordinated, available through a single proposal system that allows access to all the structural biology beamlines through a variety of mechanisms including Rapid Access proposals and Block Allocation Groups. The combination of advanced synchrotron techniques we are able to support is unrivaled for biomedical and biological research at any other US National Laboratory. Right now these beamlines represent about 34% of the users for NSLS-II. In addition to the structural biology resources described here, the CBMS also supports environmental and life-science operations at the NSLS-II imaging beamlines.

The manuscript is organized in the following fashion. Firstly we present a brief history of the structural biology programs at our predecessor facility, the NSLS; we then outline the resources available for structural biology researchers at NSLS-II; this is followed by a sketch of our plans for micro-focusing, automation, multi-modal research and computing developments.

## 2. A brief history of structural biology at NSLS

The US Department of Energy funded construction of NSLS at BNL during the late 1970s, achieving first light in 1982. It was the first dedicated synchrotron light source in the United States. During its construction, aware of developments for MX at SSRL and in Europe, BNL scientists B. Schoenborn, J. Hastings and W. Thomlinson proceeded to develop dipole-based beamlines. The first were small-angle X-ray scattering (SAXS) at X12-B and MX at X12-C, developed by M. Capel and R. Sweet, respectively. A summary of this and later work is provided in Table 1 ▸.

A range of other dipole beamlines supporting biological X-ray applications appeared during the late 1980s and early 1990s: X26-C supported by the Consortium for Advance Radiation Sources led by K. Moffat (and several years later by Cold Spring Harbor laboratory, Stony Brook University and the Georgia Research Alliance led by D. Schneider), X8-C by Argonne National Laboratory led by E. Westbrook (and several years later by Los Alamos National Laboratory, UCLA and Hoffman–La Roche led by J. Berendzen), and X9-B by the University of Pennsylvania led by B. Chance and K. Blasie for biological X-ray absorption spectroscopy and X-ray scattering (and several years later for MX by the US NIH and Albert Einstein College of Medicine, later Case Western Reserve University led by A. Wlodawer and M. Chance). In the early 1990s, W. Hendrickson developed X4-A and X4-C with Howard Hughes Medical Institute funding, led by Craig Ogata. In the late 1990s, led by S. Burley and M. Chance, Rockefeller University, Memorial Sloan-Kettering Institute, Weill Cornell Medical School and Albert Einstein College of Medicine (later Case Western Reserve University) started an MX program at beamline X9-A. In the early 2000s, NIGMS funded the construction and operation of the X6-A beamline, an MX beamline at the NSLS (Stojanoff *et al.*, 2004 ▸); X6-A was the first funded dipole beamline operated and managed by the NSLS facility.

By 2000 the NIH supported several MX programs including this X6-A beamline. Most of the remaining MX beamlines were included in the PXRR (Macromolecular Crystallography Research Resource) effort starting in 1999. This funding was joint from programs at NIH (NCR) and the Biological and Environmental Research arm of the DOE, who provided funds to the Biology department and then NSLS and NSLS-II. This program employed about 20 people; it was created by R. Sweet, M. Capel and L. Berman, and was directed by R. Sweet until the closure of the NSLS.

Later developments included biological X-ray scattering capabilities led by L. Yang first at X21-A1 and later at X9 in 2006–2007 on a dedicated insertion device beamline. Synchrotron XFP was developed first at the X9-A dipole beamline in the late 1990s, and at X28-C 2000 onwards, led by M. Chance (Albert Einstein College of Medicine and later Case Western Reserve University).

Insertion-device beamlines for MX started with the X25 wiggler in 1990, stimulated by J. Hastings and led by L. Berman and R. Sweet, funded by the DOE. This beamline was important in the work of three Nobel Prize winners: R. MacKinnon, V. Ramakrishnan and T. Steitz. Then, in the early 2000s, E. Johnson, M. Chance and R. Sweet created the X29 undulator beamline. This was operated by W. Shi and H. Robinson, becoming a huge producer of new structures. Eventually, in about 2003, we developed funding to replace the X25 wiggler by a very powerful undulator, installed in 2006. These beamlines remained remarkably productive contributing to a sum total 400–500 PDB depositions per year to the end of the NSLS operation in 2014.

Training was always a major part of the NSLS philosophy. The X6-A program team developed the MX workbench training concept and the crystallization-focus training. These events were directed towards early career faculty and researchers and helped many get started. The workbench concept was adopted by the SAXS, X9 beamline and continues now, operated by the CBMS as a portion of the NSLS II Structural Biology effort. The NSLS also developed the RapiData Course (Sweet & Soares, 2010 ▸) which ran annually from 1999 to 2014 and focused on the synchrotron-based component of modern MX (this program continues with this name and same format at SSRL.)

NSLS contributed strongly to the justification for development and construction of NSLS-II in several ways: methods and instrumentation of course, but more important was providing the basis for spectacular growth of the community of structural biologists in the Northeastern United States. Throughout the period described, MX benefited from the support of the biological and environmental research arm of the DOE and the NIH (NCR, NCI and now NIGMS).

## 3. Structural biology beamlines at NSLS-II

**Figure 1:**
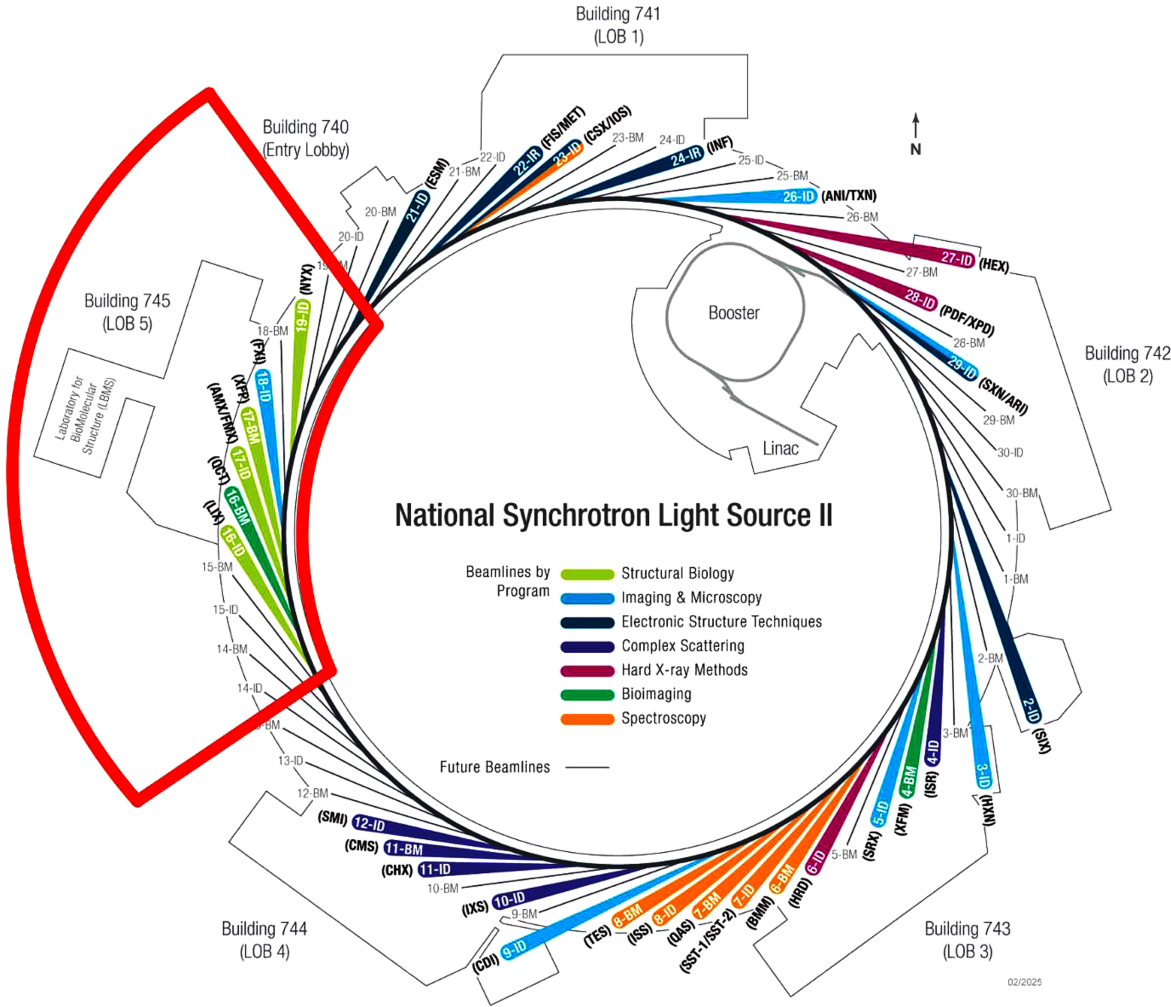
Symbolic map of NSLS-II showing the locations of the structural biology beamlines that form the CBMS (LiX, AMX, FMX, XFP), along with the partner beamline NYX. Laboratories to support the scientific programs on the beamlines are conveniently located in building 745.

**Figure 2:**
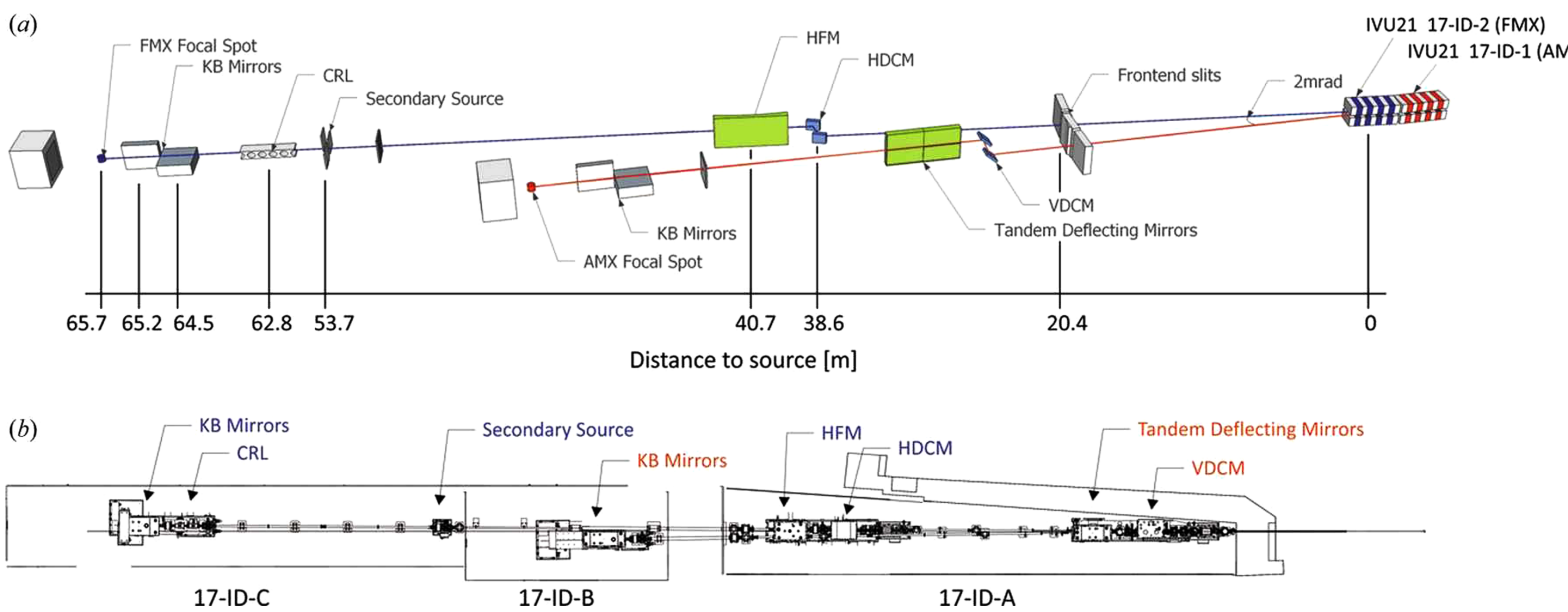
(*a*) FMX and AMX beamline layout (not to scale). AMX is served by the upstream (17-ID-1) IVU21 undulator, FMX by the downstream (17-ID-2) one. Beam and labels of AMX are red, those of FMX are blue. All distances are given with respect to the FMX source position. (*b*) Top-down plan view of the beamline layout on the NSLS-II experimental floor. The white-beam path to the monochromators is contained in the 17-ID-A lead hutches, followed by the AMX experimental hutch 17-ID-B and the FMX experimental hutch 17-ID-C. Figure taken from Schneider *et al.* (2021 ▸).

All the beamlines we will describe are built in the same area of the NSLS-II experimental hall, making common facilities to support all experiments and experimenters readily accessible (Fig. 1 ▸). The beamlines FMX and AMX share the same sector 17-ID and have been described in detail elswhere (Schneider *et al.*, 2021 ▸; Schneider *et al.*, 2022 ▸). AMX and FMX are fed by canted undulators with 2 mrad separation; their beams come out close together, sharing the X-ray-optics hutch (Fig. 2 ▸). Each has a double-crystal monochromator and mirror optics to deliver the X-ray beams, with minimum dimensions (H×V) of 1.5 µm × 1.0 µm for FMX and 7 µm × 5 µm for AMX. AMX keeps the beam size fixed at its minimum size, where FMX allows users to instantly expand the beam to 3 µm × 5 µm (default) and 10 µm × 10 µm by inserting Be lenses. They have experimental stations with high-quality X-ray optical systems to pro­duce a conditioned beam, and mech­anisms with air bearings in critical places to orient the specimen crystal in those beams. Both have Dectris Eiger area detectors for rapid, high-efficiency diffraction data collection: FMX has the E16M framing at 133 Hz; AMX has the E9M framing at 238 Hz.

**Figure 3:**
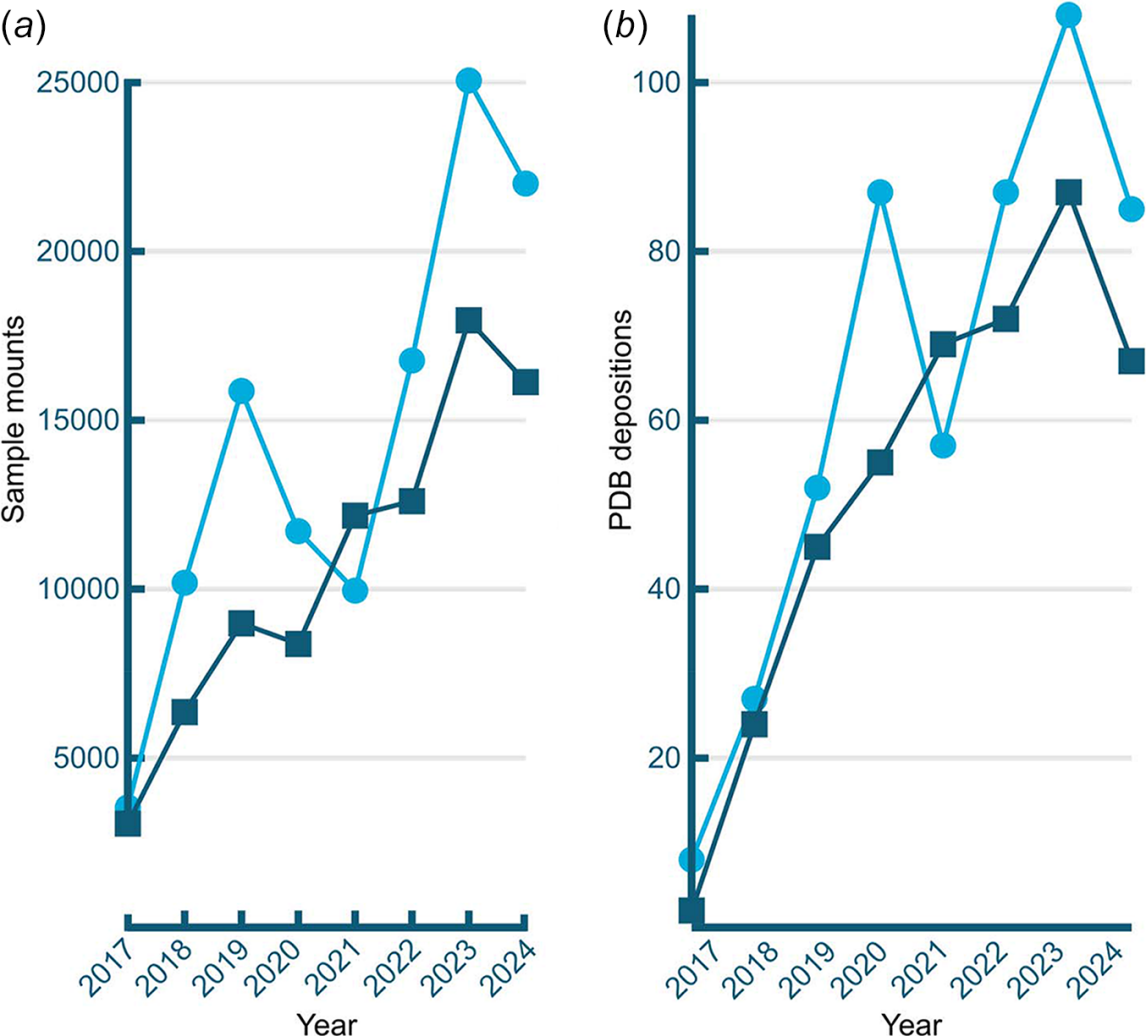
(*a*) Annual tallys of the number of samples mounted at AMX (cyan, round points) and FMX (dark blue, squares) beamlines. (*b*) Depositions in the Protein Data Bank per year (same color scheme).

Each has a Stäubli six-axis robot with NSLS-II pneu­matic gripper to mount crystals on Spine caps from UniPucks (Lazo *et al.*, 2021 ▸). The automated cryogenic supply chambers each hold 24 UniPucks, for a total load of 384 specimens. The high-brightness microfocus beams, together with real-time spot-finding analysis, enabled ultrafast rastering, where the crystal is scanned across the beam in two directions to obtain a heat map of the best diffracting volumes within seconds. This is the basis of our success in locating the best diffracting crystal volumes for the highest-quality structures, the full automation of the data-collection workflow and obtaining structures from previously intractable crystals, and showing strong year-on-year growth (Fig. 3 ▸).

The scattering beamline LiX occupies sector 16-ID (Yang *et al.*, 2020 ▸), fed by a single undulator. A double-crystal monochromator, mirror optics, a fixed secondary source and compound refractive lenses provide a range of beam dimensions from a few micrometres to a fraction of a millimetre. The LiX beamline was designed to be flexible, accommodating a wide range of experiments. However, to make the best of limited resources, we primarily focus on two types of experiments, solution scattering (Yang *et al.*, 2020 ▸) and microbeam structural mapping (Yang *et al.*, 2022 ▸), with dedicated staffing to help users optimize experiments and the resulting data.

The solution-scattering operation is automated using a six-axis robot and standardized sample holders. A popular option is to provide in-line size-exclusion chromatography (Byrnes *et al.*, 2021 ▸). A completely different measurement is to scan a tissue specimen on a solid support to create structural maps based on the information extracted from the observed scattering pattern at each point on a grid (Yang *et al.*, 2022 ▸). Two detectors collect small-angle and wide-angle scattering data.

The synchrotron XFP beamline is located in the 17-BM sector, adjacent to FMX and AMX. This beamline uses a three-pole wiggler source and a front-end focusing mirror optic to deliver broadband pink beam (*E* = 4.5–16 keV) to the sample (Asuru *et al.*, 2019 ▸). The beamline provides access to a relatively unique (only one other such beamline worldwide, at the ALS) solution-state probe of biomolecular structure, in which broadband ionizing radiation activates water molecules in solution. These generate hydroxyl radicals that can then covalently label biomolecules on microsecond to millisecond timescales.

Most experiments are performed in a partially defocused configuration with a beam size of several millimetres, using a high-throughput 96-well apparatus that supports steady-state and freeze-quench samples at a range of temperatures (Jain *et al.*, 2021 ▸). This has been applied to studies of, among other things, prion proteins, virus assembly and small-molecule drug binding. Other experiments, such as time-resolved studies (Du *et al.*, 2019 ▸) or very high X-ray dose, can be performed in a flow configuration at the beamline’s 1:1-focus position using a beam size of 100 µm × 400 µm. This native solution-state method solves cutting edge problems in protein and nucleic acid interactions and dynamics, complementing and extending the suite of state-of-the-art biophysics capabilities in CBMS. The FP program is enhanced by access to mass spectrometry on-site at the NSLS-II near the XFP beamline, improving accessibility of the method to the user population, particularly for new investigators.

## 4. Microfocusing crystallography at AMX and FMX

The AMX and FMX beamlines provide the brightest microfocus beams available to the MX community in the US, and only now do a few operational MX beamlines, for example at the upgraded ESRF synchrotron source, surpass these beam properties – with a pink beam and an updated lattice, the EBSL8 beamline’s flux density is two orders higher than FMX’s. We will outline how these beams are ideal for collecting data from smallest microcrystals, and from challenging and irregular large crystals, to support structure determination from challenging macromolecular targets. We give examples in Section 4.1.

The *Life Sciences Data Collection Graphical user interface*, *LSDCGui*, is used to collect data at the three MX beamlines. The data collection GUI heavily relies on NSLS-II standards and packages [*EPICS*, *ophyd*, *bluesky* (Allan *et al.*, 2019 ▸), *MongoDb*] to orchestrate data collections using pre-determined protocols (Schneider *et al.*, 2021 ▸; Schneider *et al.*, 2022 ▸). Today, LSDC is supplemented by a puck-importer application that takes a user-provided sample spreadsheet, curates it and submits the required collection requests to LSDC queuing. In manual operation, LSDC allows users to perform collections from a wide array of protocols including rastering, standard, vector, multiple collections and more. Critical underlying applications were updated to rely on recent advances in AI/ML, such as the main sample centering routine, which uses a neural network that was trained on a wide variety of sample pins and loops to detect the loop outlines and, where possible, the crystals to set up diffraction-based raster screens.

The core capabilities for both beamlines, next to high beam- and sample-stability, and high image quality high-resolution crystal-alignment microscopes, are advanced data-collection protocols. We establish every diffraction data collection at our beamlines as a highly optimized vector-scan protocol. This may result in data collection with no translation of the crystal, to helical scans that help distribute the X-ray dose over a larger volume crystal, to vector-stacks for obtaining ultrafast diffraction heat maps. This versatility readily enables location of the best diffraction volume of a crystal, and then using it in the optimal way.

We have established a suite of tools – outlined in the following paragraphs – to help users make best use of this beam to obtain better data from more – and from more challenging – samples. We make the X-ray beam properties accessible by automating data collections to the greatest extent possible, by providing a carefully chosen selection of data-collection protocols and processing workflows. Based on the experience derived from thousands of data collections, we offer a selection of parameters like energy, beam size and temperature to cover non-standard conditions for advanced experiments.

### 4.1. The promise of high-flux microbeams: applications

MX beamlines with bright microfocus beams excel at collecting complete datasets from the smallest microcrystals. Early crystallization hits are often only a few micrometres in size; we can now collect data from these, where previously the only option was to continue crystal optimization. In a recent example, a 360°, 90 µm-vector collection of a 5 µm thin needle crystal at the FMX beamline yielded a structure of a KRAS peptide bound to a human leukocyte antigen relevant to cancer therapy research (Wright *et al.*, 2023 ▸). Multi-crystal datasets can extend to over 1000s of microcrystals for a SAD dataset from crystals under 10 µm in size (Guo *et al.*, 2019 ▸).

For large crystals, microfocus beams are advantageous because they enable the collection of high-quality structures from inhomogeneous crystals, like a 2.7 Å riboswitch structure obtained with a 1 µm beam from several 2 µm-wide crystallites of an RNA crystal agglomerate (Peselis & Serganov, 2018 ▸), or from crystals that grow in clusters or stacks of microcrystals, such as SARS-CoV2 main protease crystals with ligands (Andi *et al.*, 2022 ▸). The microfocus beam of AMX was used to probe the local occupancy of a soaked ligand in a large crystal to obtain a high-occupancy structure from a partially soaked crystal (Schneider *et al.*, 2022 ▸).

### 4.2. Data collection strategy

At the core of our beamlines’ capabilities is the ability to collect diffraction-intensity heat maps within seconds. By providing precise information about both the location of smallest microcrystals and their diffraction quality, ‘diffraction rasters’ have become our everyday workhorse, used for every crystal that is measured at the beamlines.

The power of these X-ray beams bring some challenges since many experimenters have not experienced the high brightness and small beam size encountered at our beamlines. To enable them to plan their experiment and dose delivered to the sample, we provide an interface to *RADDOSE-3D* (Dickerson *et al.*, 2024 ▸), which derives an estimated sample lifetime with a conservative default choice of experimental parameters. For detailed dose-planning, for example for room-temperature experiments, large unit cells, sensitive metal centers or tailoring for a specific sample, our GUI for *RADDOSE-3D* enables users to choose LSDC exposure parameters, and customized PDB files, for a range of crystal sizes. The interface includes 3D volume plots of the dose isosurfaces of the beam sweep in the crystals for complex geometries.

### 4.3. Multi-crystal/serial data collection and sample delivery

For small crystals, the available crystal volume limits the number of frames one can take from a single crystal (Holton & Frankel, 2010 ▸). To obtain complete datasets, data from multiple crystals then need to be merged, ranging from a few to hundreds of crystals, depending on crystal size, radiation sensitivity and crystal homogeneity. At room temperature, crystals are particularly susceptible to radiation damage (de la Mora *et al.*, 2020 ▸). We therefore offer multi-crystal and serial crystallography for our sample delivery methods, our data collection protocols, and processing pipelines. In a recent example, Gabelli and coworkers used multiple crystals ranging in size from 10 to 13 µm, and mounted in a single loop, to obtain a multi-crystal dataset of a phospho­lipid phosphatase that is commonly mutated or silenced in cancer (Dempsey *et al.*, 2021 ▸).

A multitude of sample-delivery methods are available for multi- and serial crystallography; different sample supports work well for different crystallographic methods. The following ones are regularly used here, with cryo-crystallography samples still the most popular method due to ease of use: unordered crystals for cryo-crystallography can be mounted in loops and on lithographically fabricated meshes (Guo *et al.*, 2018 ▸, Guo *et al.*, 2019 ▸). Sleeved pins (Ebrahim *et al.*, 2022 ▸) and sealed membranes (MiTeGen SSX system) work for multi-temperature data collection to ‘room temperature’ and physiological temperatures. Oxford Photochip silicon holders (Mehrabi *et al.*, 2020 ▸) are ideal fixed targets for ordering micro-crystals in regular grids for serial still-image exposures, thereby enabling targeted reaction initiation using droplets or lasers for time-resolved data collections.

A protocol named *multiCol* in LSDC automates multi-crystal data collection. It picks prioritized positions for small-wedge rotation collections from a low-dose raster scan, with a guaranteed minimal distance between the selected centers to prevent overlapping and to manage radiation damage. For harder-to-collect samples such as clusters of needle-like crystals, the *WYpeline* protocol (Gao *et al.*, 2018 ▸) collects data in a serial mode where indexable parts are identified and clustered at the processing step, avoiding the need to align a single needle hidden in a cluster.

Several software packages are available for AMX and FMX for scaling and merging multi-crystal datasets, including *XSCALE* (Kabsch, 2010 ▸), *BLEND* (Foadi *et al.*, 2013 ▸), *DIALS* (Gildea *et al.*, 2022 ▸) and *PHENIX* (Liebschner *et al.*, 2019 ▸). An in-house-developed data processing pipeline, *PyMDA*, for assembling a multi-crystal dataset (Takemaru *et al.*, 2020 ▸), features clustering capabilities for merging microcrystal sub-datasets, and was previously used to assemble diffraction datasets from 1400 microcrystals collected under cryo-conditions at long wavelengths (Guo *et al.*, 2019 ▸).

### 4.4. Multi temperature MX

The FMX instrumentation supports multi-temperature crystallography, and room temperature crystallography with humidity control, to investigate protein flexibility and dynamics. Functionally important conformations that are masked at the cryogenic temperature can be revealed by shifting temperature in multiple temperature crystallography. A series of high-resolution crystal structures of unliganded SARS-CoV2 main protease (MPro) were obtained across multiple temperatures from cryogenic to physiological temperatures (100 K to 310 K), as well as at high humidity (Ebrahim *et al.*, 2022 ▸). A multi-conformer analysis of these structures revealed a temperature-dependent conformational landscape for Mpro. This study was the first structural analysis of Mpro at human body temperature (37°C) and identified subtle conformational changes and increased flexibility in specific regions of the enzyme which are crucial for understanding its catalytic mechanism and interactions with potential inhibitors.

### 4.5. Time-resolved MX

**Figure 4:**
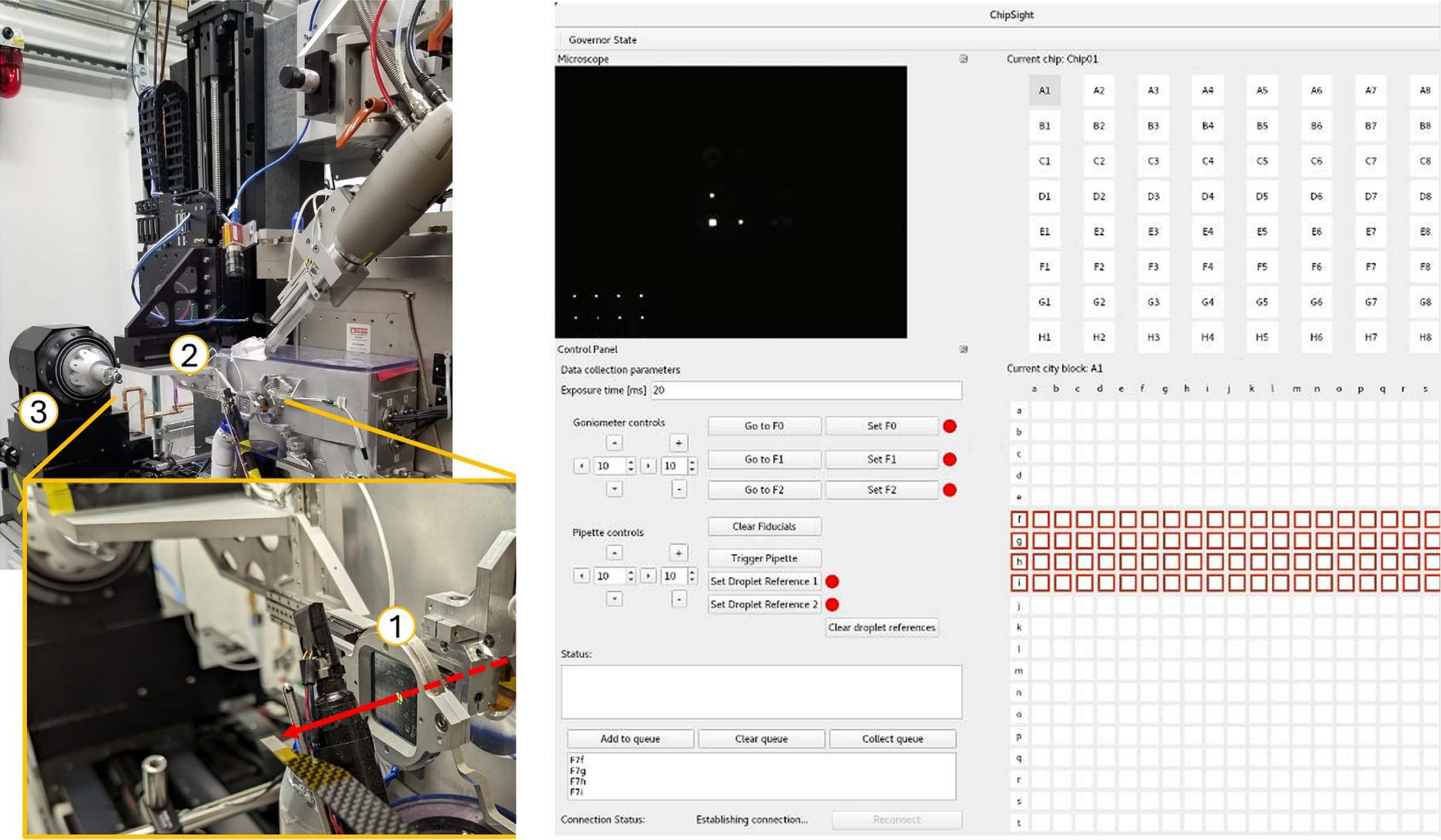
(Left) Chip scanner with a loaded Oxford Photochip (1) on the FMX secondary goniometer (2) for serial data collection at room temperature. The switch to the main goniometer (3) takes 15 min. (Right) *ChipSight* GUI for data collection with the 25600 well photochip. The microscope image shows the chip’s fiducial marks.

The CBMS supports a capability for serial synchrotron crystallography at room temperature using fixed targets on a silicon chip (Fig. 4 ▸). This will serve as the basis for time-resolved crystallography using chemical triggering. We are currently commissioning a piezo pipette for droplet delivery to initiate chemical triggering; this will allow pump/probe measurements with a time-resolution down to a millisecond. The average enzyme reaction rate is 1/(70 ms) and only few systems are faster than 1/(10 ms), so with this time resolution we will open the majority of enzymatic reactions for investigation (Bar-Even *et al.*, 2011 ▸).

### 4.6. Long-wavelength crystallography for metalloproteins

The FMX microbeam is tunable to a photon energy as low as 5 keV, useful for identification and location of functionally relevant heavy atoms in the target proteins, and for anomalous data collection for phasing. Many of our user groups study metalloproteins (Lachowicz *et al.*, 2021 ▸; Wu *et al.*, 2024 ▸; Grosjean *et al.*, 2024 ▸) containing functionally relevant metal cofactors. In some cases, groups use metal substitution to modulate the catalytic activity of enzymes, which requires characterizing the chemical nature of the active site metals using anomalous scattering. For most sensitive measurements at the long wavelength end, an He-path is available (Karasawa *et al.*, 2022 ▸).

## 5. Automation as an enhancer of science

We are witnessing a gradual, seemingly inexorable shift in user synchrotron experience. Most users of the resources employ a wide array of techniques and tools, and the crystallographic result from MX is often only one part of their investigation. Our past experience is that users of our MX beamlines have been trained and are able to handle all aspects of MX workflows, and group leaders would accompany their students to teach them. However, the pandemic of 2020 changed this.

We had actually already been working to prepare our beamlines for remote operations, so we were well placed to operate with experienced workers doing much of the crystallographic work from home while our staff performed the hands-on work at the beamline. A consequence of this is that now many experimenters have never visited a CBMS beamline nor seen the details of how the experiment works. Unfortunately the experimenter at the other end of the ‘Chat’ session is not at all an expert in this technique.

Fortunately, we had designed the AMX and FMX beamlines to facilitate the most challenging crystallographic experiments, requiring access to micro-focusing beam and high-throughput operation. So we sequentially upgraded hardware, software and methods to improve beamline performance further, and to accommodate this new paradigm: the real expertise is at the beamline, and the interested scientist is remote. With supplemental funding from the NIH we have increased user throughput while simultaneously increasing ease of operation through better automation and faster feedback (Fig. 3 ▸).

To achieve these twin improvements, the CBMS has developed the hybrid crystallographic data-collection mode. In this, the night before a beam time run, an automated (user-defined) data collection or a diffraction-quality test (raster scan only) is performed on all samples. Our staff will have mounted the user’s crystal cassettes (pucks) in the robot’s sample-delivery dewar, and the users will have submitted a spreadsheet that specifies which of these options to perform. Through importing this spreadsheet, the LSDC data collection suite queues the automated collection and staff initiate the collection. On the day of beam time, and working remotely, each of up to three groups a day has three further hours of beam time to ‘manually’ collect datasets on as many samples as needed, informed by an experimental report from the night’s automated work.

This new access mode optimizes many aspects of the experiment and relies on advanced automation. Users may benefit from automated collection for standard experiments with samples as small as 25 µm, and then can focus their time on collecting data from more challenging samples.

In the sections below we describe the improvements that achieved our current results and improvements we plan for the next five years.

### 5.1. Sample automation

Many scientific projects require screening a substantial number of crystals. In the past, most institutions leveraged in-house X-ray sources to screen early crystals locally. This is no longer true; most institutions no longer operate X-ray sources, but the need for early screening remains certain. The AMX and FMX beamlines rely on a high-capacity sample changer holding 384 Spine bases in liquid nitro­gen and a six-axis robotic arm fitted with NSLS-II-designed gripper. A reliable sample-exchange time of 35 s allows for continuous overnight automated operation, allowing rapid screening of the whole dewar in under 10 h. The sample automation developed is compatible with the higher density format of miniSpine, in the event that format is adopted.

### 5.2. Automated crystallographic data collection

For automated data collection, one central application is the loop centering, which is now based on detectron2 (Meta), an object-detection model that was trained on AMX on-axis microscope images. The new model detects the loop, aligns the loop ‘face on’ and automatically draws the two orthogonal rasters with optimized dimensions. These two orthogonal rasters are used to align the sample optimally, ready for standard collection. We plan to improve the model further to detect crystals using a large training set. If the first raster does not detect diffraction, the second will be skipped, and no data will be collected. This contributes to a significant improvement in achievable throughput; we now routinely collect from 24 samples per hour on AMX, a 50% improvement when compared with previous loop-detection and centering.

This new workflow, loop detection plus two orthogonal raster scans is also employed for manual collection, and is the base for future improvements, to include automated vector collection and multiple standard collections. Users may focus now on rapid rastering analysis to best center samples before data collection is launched. We are also developing new scoring functions to optimally highlight or select the best diffraction regions of the samples for manual and for automated collection.

Our goal is to enable automated optimal collection from at least 95% of all samples passing through our beamlines. Expert or trained users will then be able to invest collection time on those outlier samples with the most challenges.

To increase reliability, we also are developing ‘sentinel’, a set of Python scripts that automatically recovers from known sets of recurring issues, allowing continuous uninterrupted overnight operation. We aim to automate more complex experiments, including both standard and vector data collection, based on the two orthogonal rasters, and on advanced analysis including indexing of spots in each image. Other improvements that might contribute to increased throughput, reliability and runtime remain to be finalized and will be described in an independent contribution.

### 5.3. Data processing and analysis

When operating in our hybrid mode, the automated data-examination workflows relentlessly labor through up to 275 samples in the 12 h of automated screening or collection. To enable the data reduction, we have a dedicated 27 node compute cluster providing 3584 cores accessible over 100 GB network links, and backed by 3.5 PB of dedicated storage. Raster data are analyzed in real time by *Dozor* (Svensson *et al.*, 2015 ▸; Zander *et al.*, 2015 ▸), providing information for sample centering. All data collected at the NSLS-II undergo data reduction to structure factors with *fast_dp* (Winter & McAuley, 2011 ▸) and academic data will include *autoProc* (Vonrhein *et al.*, 2011 ▸) results too. Academic users are encouraged to provide PDB models, two dimple (Winn *et al.*, 2011 ▸) instances are executed, one per data reduction method. This considerably speeds up overall analysis to enhance the scientific outcome. Only a handful of datasets will then be selected for structural analysis and manual processing for PDB deposition supporting scientific results. We are developing additional tools to support research requiring hierarchical cluster analysis (Nguyen *et al.*, 2022 ▸) and to implement, in collaboration with others, additional automated analysis in downstream steps, model building/refinement and ligand binding.

## 6. Science areas benefitting from and enabled by these advances

### 6.1. High-throughput fragment screening

Fragment-based drug discovery is an increasingly important technique for developing lead compounds in early phase drug discovery. Small (<250 Da) chemical fragments can sample chemical space much more efficiently than larger, more targeted molecules (Krojer *et al.*, 2020 ▸). In turn, fragment scaffolds can be rapidly extended and modified with purposefully designed building-block libraries that are readily available from multiple vendors. As of 2024, as many as 50 compounds revealed by this method are in clinical trials including seven drugs with FDA approval (Nature Fragments in Drug Discovery). The difficulty resisting truly efficient sampling is that fragment compounds tend to bind with low affinity (*K*_d_ ≃ m*M*), so this requires development of increasingly sensitive analytical techniques for binding characterization (Schiebel *et al.*, 2016 ▸).

Advances in automation and data-collection rates at MX beamlines have enabled X-ray crystallographic fragment screening (XCFS) as a viable technique for performing primary screens. At NSLS-II, in response to a growing demand from our user community, we have implemented a pilot XCFS user program modeled after that of the XChem facility at the Diamond Light Source (Oxford, UK). Both use acoustic liquid handling to transfer fragment compounds to crystallization plates, followed by manually assisted crystal harvesting (Douangamath *et al.*, 2021 ▸). Users need only provide high-quality crystals (<2.5 Å diffraction), grown in their home laboratories or on-site at NSLS-II. Our facility provides libraries and other paraphernalia. Since our initial pilot project in 2023 the facility has screened seven targets corresponding to approximately 6600 crystals. X-ray data collection is completely automated at a collection rate of approximately 500 samples per day with raster-based crystal centering.

### 6.2. Development of next generation advanced analysis using supervised and unsupervised AI and ML, and NSLS-II-generated and curated data

The 2024 The Nobel Prize in Chemistry was awarded to David Baker ‘for computational protein design’, and to Demis Hassabis and John M. Jumper ‘for protein structure prediction’. These two 2024 articles (Krishna *et al.*, 2024 ▸) by David Baker’s group and (Abramson *et al.*, 2024 ▸) by Hassabis and Jumper’s, detail the recent state of the field. The advances this represents have changed our lives at MX beamlines. In the early days, starting at NSLS about 1984, the principal focus was on measuring phases, ultimately to solve the protein crystal structures. But from that point, and then increasing over the decades, structures began to appear in the Protein Data Bank that were related to some new target, so one could use the molecular replacement (MR) method to fit the new molecule into its diffraction data: no need to find phases.

This has all come together in recent years and depends on the database produced by the UniProt Consortium, a collaboration among the European Bioinformatics Institute (EBI), the Protein Information Resource and the Swiss Institute of Bioinformatics. It contains about 250 million protein sequences, and other functional annotation. The *AlphaFold* structure-prediction software system was created largely by Hassabis and Jumper, in a partnership of *Google DeepMind* with EMBL’s European Bioinformatics Institute (EMBL-EBI). This software was applied to the sequence database to produce the *AlphaFold DB*, which offers a prediction of what each of the over 200 million known proteins in *UniProt* might look like.

The implications for us at the beamlines is that now we have experimental pipelines that, of course, do no work to measure phases, but immediately will employ MR to fit the appropriate *AlphaFold* model into the data we measure, thereby producing an experimentally precise model!

These advances are all-atom predictions that remarkably can generate protein–ligand complex structures from protein sequences and molecular graph representations. But significant challenges remain to capture the full complexity of these interactions. These shortcomings may be due in part to a lack of salient high-quality training data, not only structural data but other pertinent physicochemical information (Baek, 2024 ▸).

High-throughput pipelines such as X-ray crystallography fragment screening laboratories, which generate large volumes of structural data, are poised to integrate these data into AI models that incorporate other sources of information. Saar *et al.* (2023 ▸) describe an early example of combining high-volume structural data with physics-inspired descriptors to generate protease inhibitors with enhanced affinity. Further investment in the technology and wetlab workflows that enable these types of examples will pay dividends.

We begin now to see the extrapolation of this: biologists begin to interpret their observations by referring to *AlphaFold* models, not to any precise crystal structure. What does the future hold for us at MX beamlines?

## 7. Data collection trends and outlook

The crystal samples driving the evolution of microfocus crystallography are shifting. The most demanding samples for micro-crystallography used to be membrane proteins grown in lipidic-cubic-phase media. Today we observe that multi-crystal crystallography using rotation collection is routinely used for smaller crystals. We expect that serial still-image data collection on fixed targets will be the main method used for time-resolved experiments.

### 7.1. Human versus machine

Microfocus data-collection strategies that were thought to require human intervention can now, or will soon be able to, run in a fully automated fashion. New workflows employing ML further accelerate this trend. Where a researcher still needs to direct data collection manually (for the time being), we facilitate their data collection by automating the intermediate steps.

### 7.2. Making microfocus crystallography more accessible

We regularly overestimate how large a crystal is needed to be able to obtain useful diffraction data. We have successfully obtained datasets from 1 to 3 µm-sized crystals. To support routine use we offer dedicated microcrystal screening protocols, where an initial estimate of the quality of microcrystals may be assessed. Where single-crystal diffraction is observed, data may be collected from optimized sample holders to collect data, or to help improve diffraction quality further.

At NSLS-II our aim is to facilitate the use of the collection workflows further through optimized user interfaces. The new way for an operator to align vector collections is by mouse-dragging the vectors on the heat maps, making the alignment faster and more intuitive. And by combining automatic raster-grid placements using ML, with the automatic microcrystal wedge rotation collection of the *multiCol* protocol, we can fully automate many multi-crystal data collections. At FMX, we allow users to change the beam size to accommodate beam times with highly varying crystal conditions. By obtaining crystal sizes from analyzing diffraction heat maps, we plan to guide users in selecting the ideal beam size for their crystals.

We expect further innovation will allow another jump to smaller accessible crystal sizes, to the ability to collect more datasets from fine features in large crystals and to achieve faster time resolution in time-resolved crystallography. New experimental protocols, and a finer high-flux beam will allow one to rapidly collect and analyze hundreds, if not thousands, of complete datasets. This will produce ensembles of crystal structures rather than simple structural snapshots, providing a mapping of the conformational space of biological macromolecules, thereby allowing access to information concerning protein dynamics. Fast data collection at room temperature using serial synchrotron crystallography enables one to capture structures of reaction intermediates. With the new beam properties, we plan to increase the accessible time resolutions to the micro-second regime, thereby to facilitate time-resolved studies of molecular mechanism and function.

## 8. Conclusions

We have presented a coordinated set of synchrotron instruments that individually make good use of the NSLS-II properties and provide a unique suite of instruments accessible through a single point of entry.

## Supplementary Material

Supporting figure. DOI: 10.1107/S1600577525003194/he5686sup1.pdf

## Figures and Tables

**Table 1:** Technology developments for MX at NSLS

Facility/activity	Description	References
NSLS dipole MX beamlines	X12-C, dipole	Helliwell (1992 ▸)
	Software development and others	Skinner *et al.* (1996 ▸); Skinner & Sweet (1998 ▸); Skinner *et al.* (2006 ▸)
	X4-A	Staudenmann *et al.* (1989 ▸); Lidestri & Hendrickson (2009 ▸)
	X6-A	Stojanoff *et al.* (2004 ▸)
	X8-C	Alkire *et al.* (1995 ▸)
	X9-A	Burley, S. & Chance M.
	X26-C	Stoner-Ma *et al.* (2011 ▸); Orville *et al.* (2011 ▸)
NSLS dipole SAXS		Allaire & Yang (2011 ▸)
NSLS insertion device beamlines	X25 and X29 for MX X9 for SAXS by L. Yang.	Héroux *et al.* (2014 ▸); Shi *et al.* (2006 ▸)
X-ray footprinting	X9-A, X28-C	Sullivan *et al.* (2008 ▸)
NSLS-II beamlines, FMX, AMX, LIX	Beamline development	Schneider *et al.* (2021 ▸); Schneider *et al.* (2022 ▸)
Laue diffraction	Capability and kinetic study	Getzoff *et al.* (1993 ▸); Singer *et al.* (1993 ▸)
Detector development	Multiple-CCD detector for MX	Strauss *et al.* (1990 ▸); Strauss *et al.* (1991 ▸); Phillips *et al.* (2000 ▸)
