# Supplementary material for: Structural biology at the National Synchrotron Light Source II
Source: J Synchrotron Radiat. 2025 Jun 26;32(Pt 4):873–83. doi: 10.1107/S1600577525003194 (PMC12236235; doi:10.1107/S1600577525003194)
Supplement: Supplementary file 1 [file s-32-00873-sup1.pdf]

JOURNAL OF SYNCHROTRON RADIATION

Volume 32 (2025)

Supporting information for article:

Structural biology at the National Synchrotron Light Source II

J. Aishima, B. Andi, L. Berman, J. Byrnes, S. Chodankar, E. Farquhar, M. R. Fuchs, J. Jakoncic, D. Kreitler, E. Lazo, S. Myers, K. Qian, R. Schaffer, V. Shekar, W. Shi, A. Soares, V. Stojanoff, R. M. Sweet, L. Yang and S. McSweeney

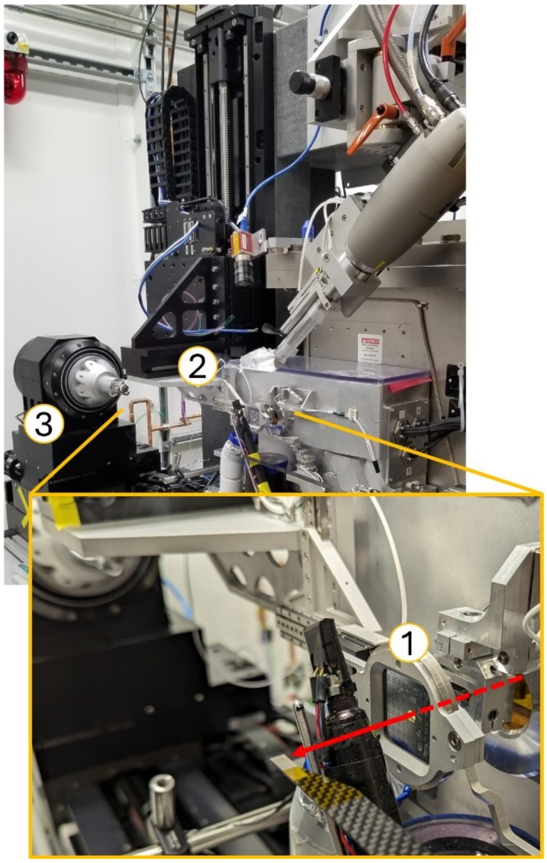

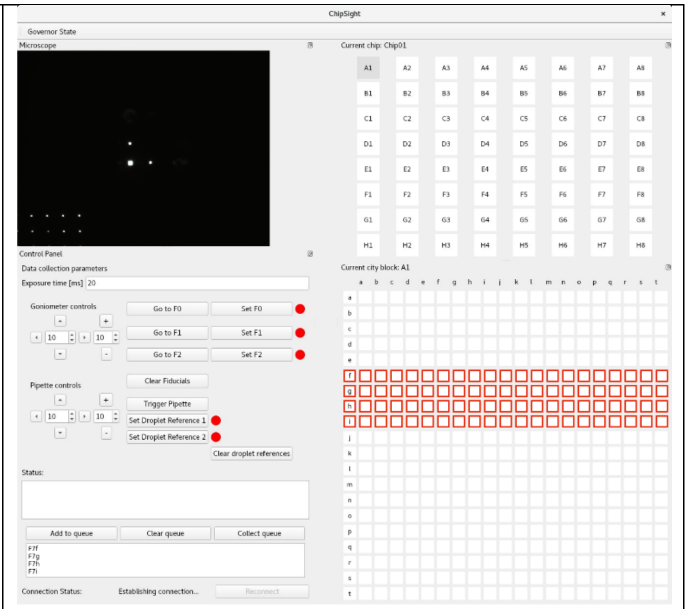

Left: The chip scanner with a loaded Oxford Photochip (1) on the FMX secondary goniometer (2) for serial data collection at room temperature. The switch to the main goniometer (3) takes 15 min. *Top:* ChipSight graphical user interface for data collection with the 25600 well photochip. The microscope image shows the chip's fiducial marks.
